# High Level Antibody Response to Pandemic Influenza H1N1/09 Virus Is Associated With Interferon-Induced Transmembrane Protein-3 rs12252-CC in Young Adults

**DOI:** 10.3389/fcimb.2018.00134

**Published:** 2018-05-15

**Authors:** Ling Qin, Dayan Wang, Dongfu Li, Yan Zhao, Yanchun Peng, Dannielle Wellington, Yanchao Dai, Huanqin Sun, Jinping Sun, Guihai Liu, Andrew McMichael, Tao Dong, Yonghong Zhang

**Affiliations:** ^1^Beijing Youan Hospital, Capital Medical University, Beijing, China; ^2^Nuffield Department of Medicine, CAMS-Oxford Center for Translational Immunology, Chinese Academy of Medical Science Oxford Institute, University of Oxford, Oxford, United Kingdom; ^3^Chinese National Influenza Center, National Institute for Viral Disease Control and Prevention, China Centre for Disease Control (China CDC), Beijing, China; ^4^MRC Human Immunology Unit, Weatherall Institute of Molecular Medicine, University of Oxford, Oxford, United Kingdom

**Keywords:** *IFITM3*, pandemic influenza H1N1, influenza, virus infection, antibody response

## Abstract

**Background:** The C allele of the interferon-induced transmembrane protein-3 (*IFITM3*) SNP rs12252, a common allele in South East Asia and China, is strongly associated with severe influenza infection. However, despite the high occurrence of rs12252-CC genotype in Chinese population (~25%), severe influenza infection is rare. The aim of study is to determine whether rs12252-CC individuals have pre-existing antibody responses to previous seasonal influenza infections.

**Cohort and Method:** A total 99 young healthy volunteers (18–20 years) were recruited and received an influenza seasonal Vaccination [A/Switzerland/9715293/2013(H3N2), A/California/7/2009 (pdm09H1N1) and B/Jeep/3073/2013-like virus (Flu-B)]. Plasma and gDNA was isolated from each volunteer before, and 14, 28, 180, 360, and 540 days after vaccination. Additionally, 68 elderlies (>65 years) were also recruited as a control group to compare the levels of antibodies at baseline between the young adults and the elderly. For each sample *IFITM3* rs12252 genotype was determined and antibody levels in response to pdmH1N1, H3N2 and Influenza B infection were measured for each time point.

**Results:** We found a significantly higher level of pre-existing antibodies to pandemic influenza H1N1/09 virus (pdm09H1N1) but not to H3N2 or FluB in CC donors in comparison with CT/TT donors prior to vaccination. No impact of *IFITM3* genotype in boosting influenza specific antibodies in young adults within 1 year after receiving seasonal influenza vaccination was observed. In addition, there was no difference in pdm09H1N1 specific antibody levels observed in the elderly cohort between volunteers carrying different *IFITM3* genotypes. Higher levels of antibodies to pdmH1N1 were observed in elderly CC carriers when compared to the young CC carriers, but this trend was not replicated in TT carriers.

**Conclusion:** *IFITM3*-rs12252 CC carriers exhibit a high level of pre-existing immunity to pdm09H1N1 compared to TT carriers in the young cohort. This suggests that compensatory mechanisms exist which might contribute to viral control in patients carrying the rs12252-CC genotype who do not become sick after flu infection. However, such a potential compensatory effect appears to be lost overtime, as evidenced in the elderly cohort. If this compensatory mechanism is lost, it may make the CC carrying elderly more susceptible to severe influenza infection.

## Introduction

Influenza remains a global threat, particularly in the elderly who are at high risk for severe infection complications such as lung disease and influenza mortality (Mertz et al., 2013). However, the mechanisms involved in how severe influenza infection develops are not fully understood (Brandes et al., 2013). We and others have shown that homozygosity for a SNP (rs12252), the C variant of the interferon-induced transmembrane protein-3 (*IFITM3)* gene, is greatly increased in frequency in patients with severe acute influenza, in particular in Chinese population where the frequency of this SNP is much higher than in Europeans (Everitt et al., 2012; Zhang et al., 2013; Wang et al., 2014; Lee et al., 2017). The mechanisms underlying this are still not clear, however it is known that *IFITM3* can restrict several virus infections in addition to influenza virus inclunding Dengue virus, human immunodeficiency virus (HIV), Hepatitis C virus (HCV) and Hantaan virus (Brass et al., 2009; Huang et al., 2011; Zhang et al., 2015). *IFITM3* rs12252-C was also shown to be associated with severe avian influenza H7N9 and Hantaan virus infections in Chinese populations (Wang et al., 2014; Zhang et al., 2015; Xu-Yang et al., 2016; Lee et al., 2017); however this association was not observed in two studies on European cohorts (Mills et al., 2014; López-Rodríguez et al., 2016) or in a recent American study (Randolph et al., 2017). Previously this SNP was suggested to generate a truncated protein with a 21 amino acid deletion in the N terminus, but this has now been shown to be highly unlikely leaving the question of how rs12252-C alters *IFITM3* still open (Everitt et al., 2012; Makvandi-Nejad et al., 2018).

Aging is associated with a gradual decline in both innate and adaptive immune functions, which may contribute to increase the risk of infectious diseases and their complications including severe influenza virus infection and a lower response to influenza Vaccination (Loubet et al., 2016). Understanding the impact of *IFITM3* variant on memory immune responses in young and elderly people will therefore be important for the development of better vaccine and therapeutic strategies in Asian populations.

In this study, we recruited a total 99 young healthy volunteers (aged between 18 and 20) who received influenza seasonal Vaccination (H3N2, pdm09H1N1 and FluB). peripheral blood mononuclear cells (PBMC), plasma and gDNA was isolated before, and 14, 28, 180, 360, and 540 days after vaccination. Additionally, 68 elderly donors (age >65 years) were recruited to compare against the young cohort. Pre-existing memory antibody responses, plasma cytokine level as well as post-vaccination antibody responses to all three vaccine strains were evaluated and compared between all *IFITM3* genotypes.

## Materials and methods

### Study cohort

A total of 150 healthy young adult volunteers (age between 18 and 20) from Capital Medical University were enrolled in the study. All volunteers had not had an influenza infection in the 6 months prior to vaccination, 99 (out of 150) were followed for over 1.5 years. A licensed Trivalent Split-Influenza Vaccine was employed, which contains A/Switzerland/9715293/2013(H3N2), A/California/7/2009 (pdm09H1N1) and B/Jeep/3073/2013-like virus (Flu-B) strain. Whole-blood specimens were collected on baseline, day 14, day 28 and month 12 and month 18 following vaccination; plasma and PBMCs were isolated and stored. In addition, 68 healthy elderlies (age >65 years) volunteers without influenza infection in the 6 months were recruited as a control group to compare the levels of antibodies on baseline between the young adults and the elderly. The study was approved by the Institutional Review Board of Beijing You'An Hospital, and written informed consent was obtained from all volunteers.

### Sequencing and genotyping of *IFITM3*-rs12252 variants

Genomic DNA was extracted from PBMCs using the PureGene DNA Isolation kit (Gentra Systems, Minneapolis, MN, USA). Genotypes of *IFITM3*-rs12252 was sequenced on an Applied Biosystems 3730xl DNA Analyser (GATC Biotech). Single-nucleotide polymorphisms were identified using Chromas (Technelysium Pty Ltd) at the BGI, Shenzhen, China as described previously (Zhang et al., 2013).

### Hemagglutinin inhibition (HI) assay and definition of protection/infection

HI assays were performed in duplicate against the influenza vaccine strains in the 2015–2016 Northern Hemisphere influenza vaccine components according to the standardized protocol by the world health organization. Seroprotection was defined as an HI titer of ≥ 40. Subsequent flu infection was defined as either the detection of influenza RNA in a swab sample by RT-PCR or a 4-fold or greater rise in HI titer, with a second titer of at least 40.

### Statistical analysis

Chi-square test (χ2) and logistic regression models were used for dichotomous variables. General linear models (GLM) were used to assess the effects of SNPs on continuous variables. We used both a mixed inheritance model (wild, mixed and mutant genotype as three categories in SNP variable) and a dominant inheritance model (wild/no wild genotypes as two categories in SNP variable) to assess the effects of SNPs. One-way ANOVA was employed in inheritance model, and the Student *t*-test was used in dominant inheritance model to evaluate the differences between CC and CT, CC and TT, CT and TT, where data were normally distributed, and Mann-Whitney test for those nonparametric analyses. Statistical test differences were considered significant if *p*-values were <0.05. Analyses were performed with the GraphPad Prism v 5 (GraphPad Software, LaJolla, CA).

## Results

### Clinical and laboratory characteristics of study subjects

A total of 99 healthy volunteers, without the experience of an influenza infection within 6 months, were enrolled in the study. Amongst them, 35 donors had the *IFITM3* rs12252-CC genotype, 36 donors had the *IFITM3* rs12252-CT genotype, and 28 donors had the *IFITM3* rs12252-TT genotype (Table 1). In addition, 68 healthy elderly volunteers (age >65 years) were recruited as a control group: 24 were rs12252-CC genotype, 32 were rs12252-CT genotype and 12 were rs12252-TT genotype (Table 2). No significant difference was observed between the rs12252 genotypes in regards to demographic identifiers (age and gender). Baseline counts of neutrophils, lymphocytes, monocytes and eosinophils amongst CC, CT, and TT donors also showed no significant differences.

**Table 1:** Characterization of the young adult cohort (age 18–20).

	***IFITM3*** **rs12252 genotype**			***P*-value**
	**CC**	**CT**	**TT**	
Total (99)	35 (35%)	36 (37%)	28 (28%)	0.056
Age (years)	18.8 ± 0.63	18.76 ± 0.71	18.45 ± 0.51	n.s.
Gender (M/F)	12/23	11/25	8/20	n.s.
	**Cell counts (×10**^**9**^**/L)**			
White blood cells	6.745 ± 0.2064	6.893 ± 0.2656	7.414 ± 0.3269	0.2022
Neutrophils	3.684 ± 0.1759	3.795 ± 0.2093	4.100 ± 0.2801	0.4141
Lymphocytes	2.542 ± 0.0881	2.523 ± 0.0961	2.756 ± 0.1320	0.2415
Monocytes	0.404 ± 0.0175	0.417 ± 0.0189	0.418 ± 0.0268	0.8745
Eosinophils	0.092 ± 0.0162	0.131 ± 0.015	0.118 ± 0.0148	0.1881

**Table 2:** Characterization of the elderly cohort (age 61–83).

	***IFITM3*** **rs12252 genotype**			***P*-value**
	**CC**	**CT**	**TT**	
Total (68)	24 (35%)	32 (47%)	12 (18%)	
Age (years)	71.5 ± 6.1	71.6 ± 5.3	68.6 ± 6.1	0.267
Gender (M/F)	10/14	10/22	5/7	n.s.
	**Cell counts (×10**^**9**^**/L)**			
White blood cells	6.687 ± 1.609	5.949 ± 1.326	6.302 ± 1.898	0.214
Neutrophils	4.001 ± 1.384	3.512 ± 1.155	4.437 ± 0.871	0.276
Lymphocytes	2.145 ± 0.853	1.976 ± 0.584	2.211 ± 0.630	0.757
Monocytes	0.462 ± 0.173	0.340 ± 0.115	0.424 ± 0.136	0.165
Eosinophils	0.154 ± 0.175	0.108 ± 0.056	0.154 ± 0.058	0.595

### High pdm09H1N1-specific antibody responses in young adults with rs12252 prior to vaccination

**Figure 1:**
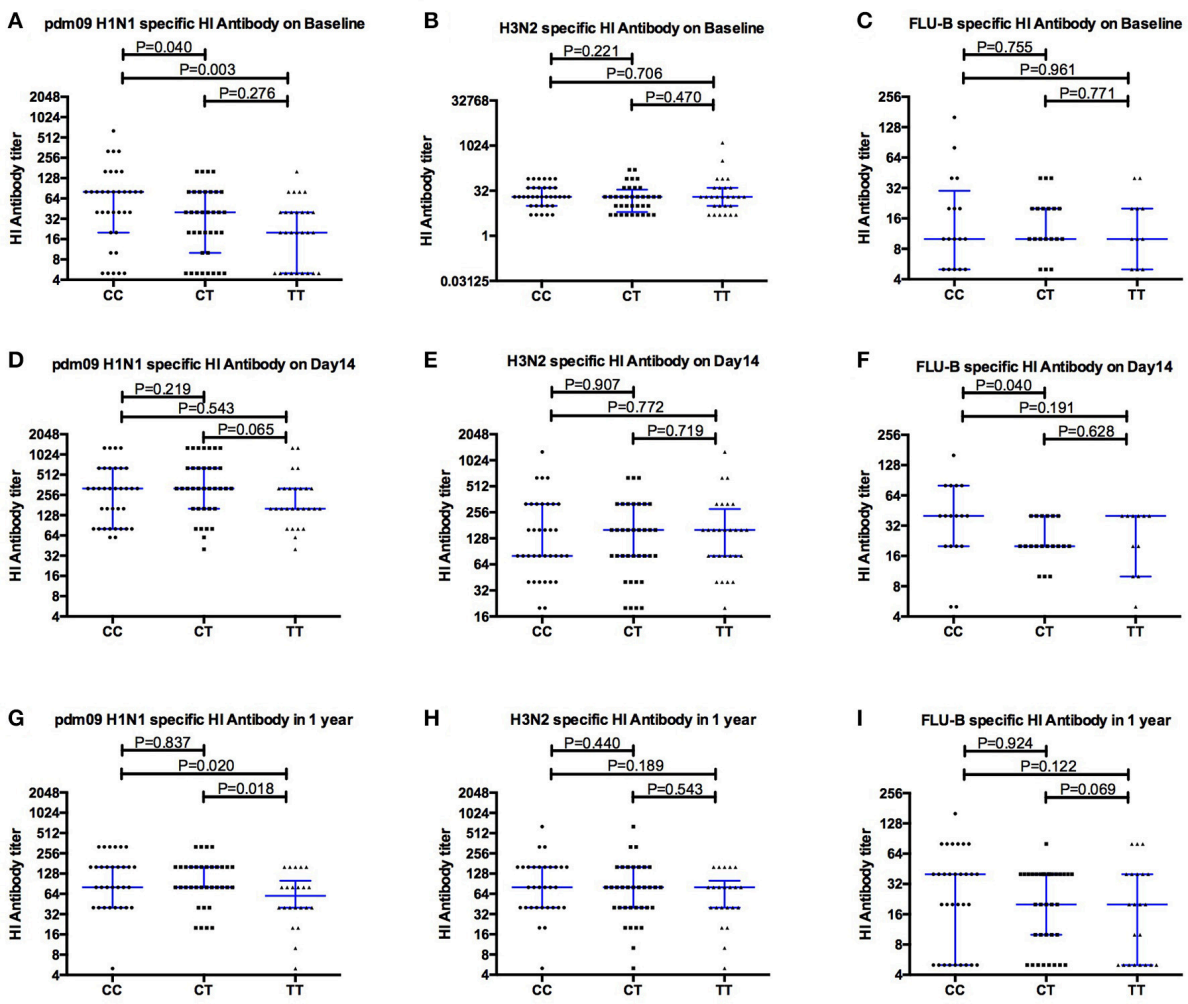
Comparing the levels of Hemagglutinin inhibition specific antibodies to pdm09H1N1, H3N2 and FLU-B before **(A–C)**, and after Vaccination Day 14 **(D–F)** and 1 year **(G–I)**, between individuals carrying different *IFITM3*-rs12252 genotypes.

Pdm09H1N1 (H1N1), H3N2 and FLU-B specific antibodies were detected at baseline to evaluate the pre-vaccination immune status of the young adult donors with different *IFITM3* rs12252 genotypes. Interestingly, the median titer of neutralizing antibody to H1N1 in donors with CC genotype was 80 (20,80), significantly higher than that in donors with CT genotype (40, *P* = 0.040) and TT genotype (20, *P* = 0.003, Figure 1A), respectively. No significant differences in antibody titers specific to H3N2 (Figure 1B) and FLU-B (Figure 1C) across each genotype were observed.

### One month after vaccination all genotypes show similar levels of antibody boost

**Figure 2:**
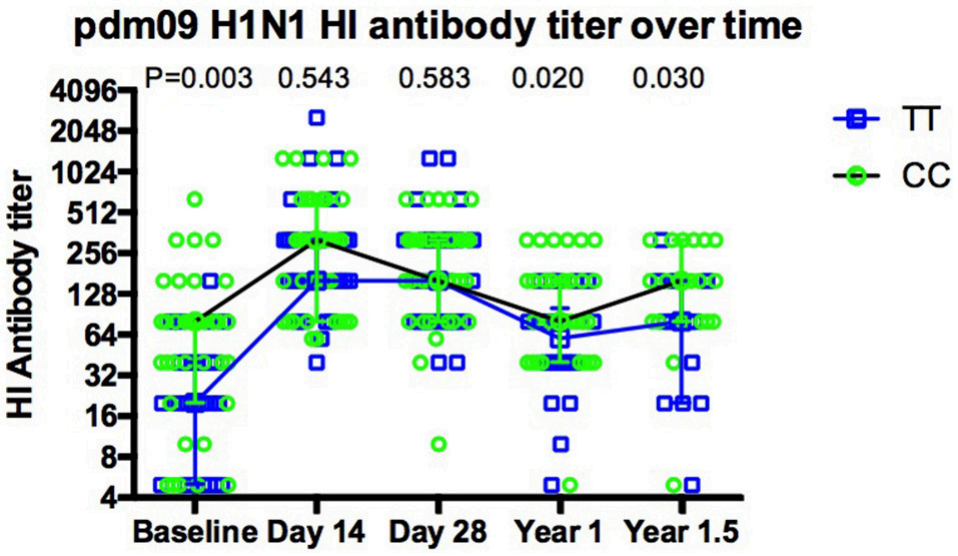
The dynamic of pdm09H1N1 specific antibodies to influenza virus before and after Vaccination. The dynamics of antibody response for CC (green circles, black line) and TT (blue squares, blue line) genotypes show that there is a more dramatic increase in TT carriers at day 14, the increase in antibodies seen at day 14 with CC carriers is not maintained and both genotypes still show a higher antibody titer 18 months post-vaccination.

As expected, overall antibody levels were elevated in the majority of the volunteers following vaccination (Figures 1D–I) at both day 14 and 1 year for all three strains. Antibody levels were comparable across all genotypes 14 days after vaccination. Geometric mean titers peaked 14 days after vaccination and the majority of individuals remained above the protection level (HI ≥ 40) to H1N1 (91%), H3N2 (85%) and FluB (50%) for up to 12 months (Figures 1D–F). Interestingly, the differences observed for H1N1 antibodies at basal level between genotypes (Figure 1A) were not observed at 14 and 28 days after the vaccination, but reappeared at months 12 and 18 (Figure 2).

### More efficient boosting of pdm09H1N1 specific antibody response in TT carriers compared to CC carriers

**Figure 3:**
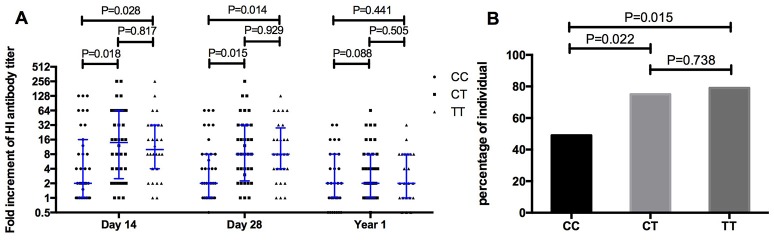
Comparing the fold increment of pdm09H1N1 specific HI antibody after vaccination **(A)** and the percentage of individuals with 4-fold increment on day 14 after vaccination **(B)** amongst *IFITM3*-rs12252 CC, CT, and TT genotypes.

The fold increase of H1N1 specific antibodies after vaccination was compared between volunteers with CC and TT genotypes (Figure 3A). The median fold of increasing antibody in volunteers with the CC genotype was 2 on both days 14 and 28, much lower than the change seen in TT genotypes (10 on Day 14, *p* = 0.028; 8 on Day 28, *p* = 0.014). Moreover, the percentage of volunteers with a 4-fold increase in antibody on day 14 was only 48.6% (17/35) in CC genotypes, much lower than that in TT genotypes (78.6%, 22/28, *p* = 0.015, Figure 3B).

### No differences in pdm09H1N1 specific antibodies observed in elderly volunteers despite different *IFITM3* genotypes

**Figure 4:**
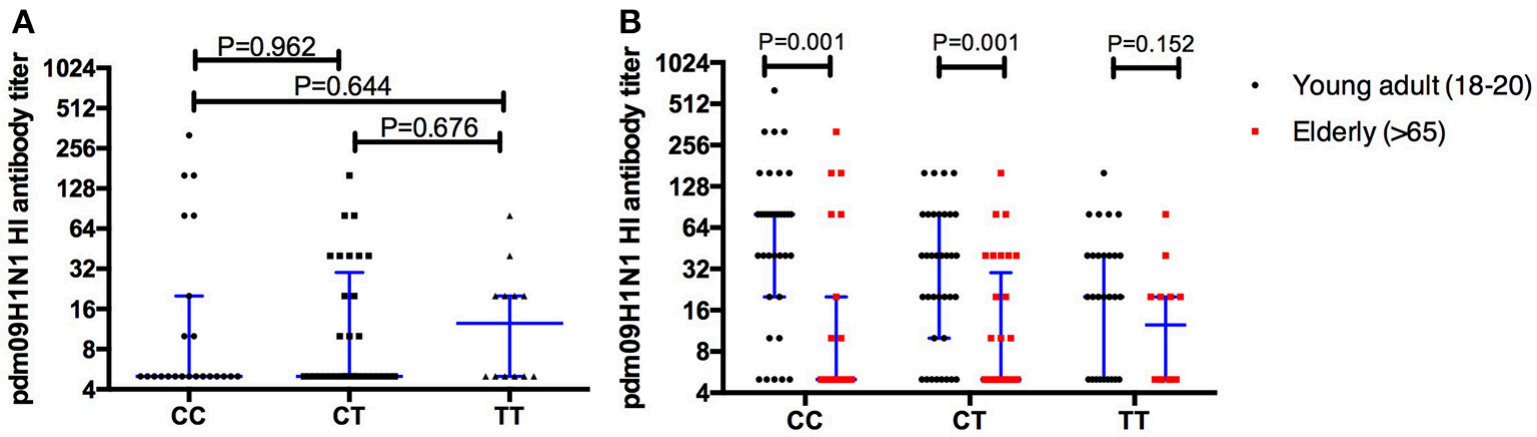
The comparison of HI antibodies specific to pdm09H1N1 between individuals carry different *IFITM3* rs12252 genotype in elderly cohort shows no significant difference between the groups **(A)**. When comparing the young adults (18–20 years) and the elderly (>65 years) the antibody titer on baseline is significantly higher in the young adults compared to the elderly with either the CC or CT genotype but not the TT genotype **(B)**.

The antibodies specific to H1N1 were evaluated in the elder population (age >65 years), and no significant differences were found between individuals carrying different rs12252 genotypes (Figure 4A). Furthermore, a comparison of H1N1 antibodies between the young adults and the elderly was performed and significant differences were found between the young and old individuals carrying CC genotype (*p* = 0.001) and CT genotype (*p* = 0.001) but not TT genotype (*p* = 0.152) (Figure 4B).

## Discussion

The rarity of severe influenza infection in China despite the high frequency of rs12252-CC, a genotype known to increase the occurrence of severe influenza infections, suggests that other compensatory factors may have a role to play in the control of this infection. We have investigated whether any differences in antibody response to various influenza strains is evident when comparing *IFITM3* rs12252 genotypes.

Our results show that *IFITM3* rs12252-CC carriers exhibit a high level of pre-existing immunity to pdm09H1N1 compared to rs12252-TT carriers, suggesting immune memory responses to pdm09H1N1 are associated with *IFITM3* genotype in young adult individuals, possibly due to more frequent exposure to influenza virus infections. In contrast, we observed no significant differences in pdm09H1N1 specific antibody levels in our elderly cohort despite comparable distribution of *IFITM3* genotypes. However, higher levels antibodies to pdm09H1N1 were observed in young CC carriers when compared to the elderly, but not in TT carriers, suggesting the loss of the potential compensatory effect in elderlies. It is worth noting that, the level of antibody to pdmH1N1 in individuals carrying heterologous genotype was significant lower than CC and higher than TT (but did not reach the significance) genotype in young adults at basal level, and there were no differences observed in elderly adults with all three genotypes. This may put older people with the CC genotype in a vulnerable position for severe flu infection, a question that merits further investigation. In addition, although the data (Figures 1A–G) suggests that antibody levels in the CT young adult carriers is always between the CC and TT levels, with a closer association to TT levels, the limited number of donors in this study prevents us from drawing firm conclusions.

It is striking to see that only pre-existing antibody responses to pdm09H1N1 were seen to be associated with *IFITM3* genotype but not responses to FLU-B or H3N2, which is in line with the current observation that *IFITM3* rs12252-C was mostly seen in association with pdm09H1N1 (Everitt et al., 2012; Zhang et al., 2013; Lee et al., 2017). A mechanistic study is needed in order to understand this potential strain specific association with *IFITM3* genotype. We speculate the potential differences between different influenza strains in association with *IFITM3* genotype could depend on sequencing variation of haemagglutinin that might affect its interaction to *IFITM3*. Additionally, the amount of infecting virus encountered at the initiation of the infection may play a role here.

We did not observe any differences in the level of boosted influenza antibody responses among young adults carrying different *IFITM3* genotypes within 1 year after receiving seasonal influenza vaccination, and antibody levels remained at a protective level particularly against influenza A virus. This result strongly suggests that *IFITM3* genotype may have little impact on the efficacy of seasonal Vaccination in young healthy adults, however its impact on live attenuated influenza Vaccine and on seasonal influenza virus vaccination of elderlies still merits a further investigation, as the potential restriction of live attenuated virus by *IFITM3* may affect the intake of the Vaccine and therefore the immune responses.

It is interesting that we have observed more efficient boosting of immune responses within one month of vaccination in TT individuals, and the levels of antibody reached a similar level as CC genotype carriers within 14 days after Vaccination. The differences between the two genotypes appeared again after 1 year of vaccination although the difference was less pronounced compared with pre-vaccination. Potentially the higher risk of influenza exposure created by having the CC genotype has resulted in more frequent exposure to influenza antigens in these individuals creating a more active phenotype in their pre-existing immune memory cells. This could be worrying for elderly CC carriers as immune responses to influenza virus could be more exhausted in these individuals due to more frequent exposure, therefore it would be even harder to generate effective immune responses to the influenza vaccine. More studies are needed to understand the impact of *IFITM3* genotype on immune memory in the older population following influenza vaccination.

## Author contributions

YoZ and TD: designed and conceptualized the project; AM and DeW: designed the experiments; LQ, DL, YP, and DeW: performed the experiments; YaZ, LQ, and TD: analyzed the data; YaZ, YD, HS, JS, and GL: provided clinical data and samples; YoZ and TD: wrote the manuscript; DeW and AM contributed in revising the manuscript. All authors have read and approved the final manuscript.

### Conflict of interest statement

The authors declare that the research was conducted in the absence of any commercial or financial relationships that could be construed as a potential conflict of interest.
